# Design of a multinational randomized controlled trial to assess the effects of structured and individualized exercise in patients with metastatic breast cancer on fatigue and quality of life: the EFFECT study

**DOI:** 10.1186/s13063-022-06556-7

**Published:** 2022-07-29

**Authors:** Anouk E. Hiensch, Evelyn M. Monninkhof, Martina E. Schmidt, Eva M. Zopf, Kate A. Bolam, Neil K. Aaronson, Jon Belloso, Wilhelm Bloch, Dorothea Clauss, Johanna Depenbusch, Milena Lachowicz, Mireia Pelaez, Helene Rundqvist, Elzbieta Senkus, Martijn M. Stuiver, Mark Trevaskis, Ander Urruticoechea, Friederike Rosenberger, Elsken van der Wall, G. Ardine de Wit, Philipp Zimmer, Yvonne Wengström, Karen Steindorf, Anne M. May

**Affiliations:** 1 Julius Center for Health Sciences and Primary Care, University Medical Center Utrecht, Utrecht University, P.O. Box 85500, 3508 GA Utrecht, The Netherlands; 2 Division of Physical Activity, Prevention and Cancer, German Cancer Research Center (DKFZ) and National Center for Tumor Diseases (NCT) Heidelberg, Heidelberg, Germany; 3 Mary MacKillop Institute for Health Research, Australian Catholic University, Melbourne, Australia; 4 Cabrini Cancer Institute, The Szalmuk Family Department of Medical Oncology, Cabrini Health, Melbourne, Victoria Australia; 5 Division of Nursing, Department of Neurobiology, Care Sciences and Society, Karolinska Institutet, Stockholm, Sweden; 6 Division of Psychosocial Research and Epidemiology, The Netherlands Cancer Institute, Amsterdam, The Netherlands; 7 R&D department, Fundación Onkologikoa, Donostia-San Sebastian, Spain; 8 Department of Molecular and Cellular Sports Medicine, Institute of Cardiovascular Research and Sports Medicine, German Sport University Cologne, Cologne, Germany; 9 Medical Faculty, Heidelberg University, Heidelberg, Germany; 10 Department of Oncology and Radiotherapy, Medical University of Gdańsk, Gdańsk, Poland; 11 Department of Laboratory Medicine, Karolinska Institutet, Stockholm, Sweden; 12 Center for Quality of Life and Division of Psychosocial Research and Epidemiology, Netherlands Cancer Institute, Amsterdam, The Netherlands; 13 Center of Expertise Urban Vitality, Faculty of Health, Amsterdam University of Applied Sciences, Amsterdam, the Netherlands; 14 Department of Medical Oncology, National Center for Tumor Diseases (NCT), Heidelberg University Hospital, Heidelberg, Germany; 15 Department of Medical Oncology, University Medical Center Utrecht, Utrecht University, Utrecht, The Netherlands; 16 Division of Performance and Health (Sports Medicine), Institute for Sport and Sport Science, TU Dortmund University, Dortmund, Germany; 17 Theme Cancer, Karolinska University Hospital, Stockholm, Sweden

**Keywords:** Exercise, Fatigue, Metastatic breast cancer, Quality of life, Randomized controlled trial

## Abstract

**Background:**

Many patients with metastatic breast cancer experience cancer- and treatment-related side effects that impair activities of daily living and negatively affect the quality of life. There is a need for interventions that improve quality of life by alleviating fatigue and other side effects during palliative cancer treatment. Beneficial effects of exercise have been observed in the curative setting, but, to date, comparable evidence in patients with metastatic breast cancer is lacking. The aim of this study is to assess the effects of a structured and individualized 9-month exercise intervention in patients with metastatic breast cancer on quality of life, fatigue, and other cancer- and treatment-related side effects.

**Methods:**

The EFFECT study is a multinational, randomized controlled trial including 350 patients with metastatic breast cancer. Participants are randomly allocated (1:1) to an exercise or control group. The exercise group participates in a 9-month multimodal exercise program, starting with a 6-month period where participants exercise twice a week under the supervision of an exercise professional. After completing this 6-month period, one supervised session is replaced by one unsupervised session for 3 months. In addition, participants are instructed to be physically active for ≥30 min/day on all remaining days of the week, while being supported by an activity tracker and exercise app. Participants allocated to the control group receive standard medical care, general written physical activity advice, and an activity tracker, but no structured exercise program. The primary outcomes are quality of life (EORTC QLQ-C30, summary score) and fatigue (EORTC QLQ-FA12), assessed at baseline, 3, 6 (primary endpoint), and 9 months post-baseline. Secondary outcomes include physical fitness, physical performance, physical activity, anxiety, depression, pain, sleep problems, anthropometric data, body composition, and blood markers. Exploratory outcomes include quality of working life, muscle thickness, urinary incontinence, disease progression, and survival. Additionally, the cost-effectiveness of the exercise program is assessed. Adherence and safety are monitored throughout the intervention period.

**Discussion:**

This large randomized controlled trial will provide evidence regarding the (cost-) effectiveness of exercise during treatment of metastatic breast cancer. If proven (cost-)effective, exercise should be offered to patients with metastatic breast cancer as part of standard care.

**Trial registration:**

ClinicalTrials.govNCT04120298. Registered on October 9, 2019.

**Supplementary Information:**

The online version contains supplementary material available at 10.1186/s13063-022-06556-7.

## Background

Breast cancer is the most commonly diagnosed cancer and the leading cause of cancer mortality among women worldwide [1]. The majority of breast cancer-related deaths are due to metastatic breast cancer (mBC) [2]. Despite the availability of a number of treatment options, treatment of mBC remains palliative with a poor median 5-year survival rate of 25% [3]. Hence, maintaining the quality of life (QoL) is one of the most important goals for patients with mBC. Side effects that affect patients’ QoL include fatigue, decreased physical fitness, insomnia, depression, neuropathy, and pain [4–8]. Of these side effects, fatigue has the most substantial impact on QoL by negatively affecting activities of daily life. Therefore, interventions that can improve QoL by alleviating fatigue and other cancer- and treatment-related side effects during palliative treatment are needed.

There is ample evidence that exercise is safe and well-tolerated during and after curative cancer treatment, and that it has a significant, positive effect on a range of side effects, including fatigue [9]. To date, these positive effects of exercise have not been demonstrated in patients with mBC, since these patients are typically excluded from exercise interventions due to the potential risk of bone fractures and poor prognosis. Only very few, mainly small studies with short interventions have been performed. A recent systematic review in patients with advanced cancer, including patients with mBC, showed that exercise is safe and feasible [10]. More specifically, it has been shown that exercise appears safe in patients with bone metastases, if it includes a supervised component [11]. The summarized evidence indicates that exercise improves physical performance and functioning in patients with advanced cancer, whereas the effects on fatigue, QoL, and other cancer- and treatment-related side effects are mixed [10]. A high-quality and adequately powered study is needed to evaluate comprehensively the efficacy of exercise in patients with mBC.

The randomized controlled EFFECT study is designed to assess the effects of a structured and individualized 9-month exercise intervention in patients with mBC on QoL and physical fatigue. Secondary aims are to investigate the cost-effectiveness of the exercise intervention and exercise effects on other cancer- and treatment-related side effects and blood markers. We also investigate the effects of exercise on overall survival, breast cancer-specific survival, and progression-free survival. Here, we describe the design of the EFFECT study.

## Methods/design

### Study design

The EFFECT study is a multinational, randomized controlled trial with two study arms: 1) the intervention arm that receives a 9-month structured and individualized exercise program in addition to usual care, and 2) the control arm that receives general physical activity advice and an activity tracker in addition to usual care, but no structured exercise program.

The study protocol was approved in October 2019 by the institutional review board of the University Medical Center Utrecht, the Netherlands, and by the local ethical review boards of all participating institutions. Written informed consent will be obtained from all participants. The study was registered with ClinicalTrials.gov on October 9, 2019 (*NCT04120298*). The first patient was included on January 8, 2020.

More information about organizational aspects of the trial can be found in Appendix I.

### Study population

We plan to include a total of 350 patients with mBC, both male and female. Patients must meet the following inclusion criteria: age ≥ 18 years; diagnosis of breast cancer stage IV; ECOG (Eastern Cooperative Oncology Group) performance status ≤ 2; and able and willing to perform the exercise program and wear the activity tracker. A patient who meets any of the following criteria is excluded from participation: unstable bone metastases inducing skeletal fragility; untreated symptomatic brain metastasis; estimated life expectancy <6 months; serious active infection; too physically active (i.e., >210 min/week of moderate-to-vigorous exercise) or already engaging in intense exercise training comparable to the EFFECT exercise program; severe neurologic or cardiac impairment according to the American College of Sports Medicine (ACSM) criteria [12]; uncontrolled severe respiratory insufficiency or being dependent on oxygen supplementation in rest or during exercise; uncontrolled severe pain; any other contraindications for exercise; any circumstances that would impede adherence to study requirements or ability to give informed consent; or pregnancy. Medical in- and exclusion criteria are checked by an involved physician at the treating hospital.

### Recruitment and randomization

**Fig. 1:**
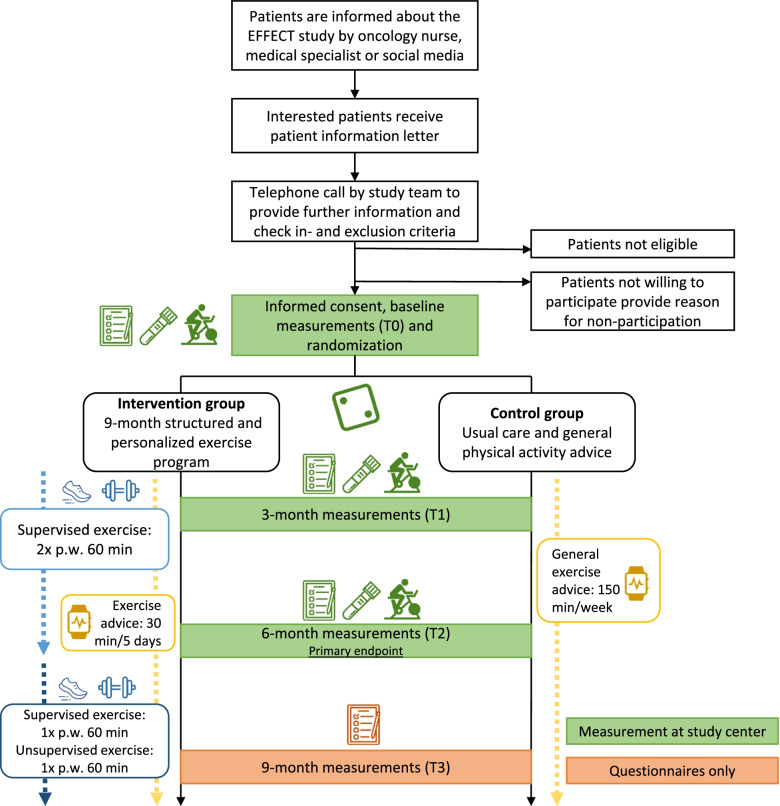
Recruitment and study procedures

Participants are recruited in Germany (Heidelberg University Hospital/German Cancer Research Center (DKFZ)/National Center for Tumor Diseases (NCT) Heidelberg and German Sport University Cologne (DSHS)), the Netherlands (University Medical Center Utrecht (UMCU) and the Netherlands Cancer Institute (NKI)), Poland (Wielkopolskie Centrum Onkologii (WCO)), Spain (Onkologikoa (ONK)), Sweden (Karolinska Institutet (KI)), and Australia (Australian Catholic University (ACU)). Some centers have invited additional recruitment sites to contribute patients to the study. The study procedures are summarized in Fig. 1. Potentially eligible patients are informed about the study by an oncology nurse or medical specialist during a regular visit or by mail/letter. In addition, social media (e.g., of national/local patient organizations) are used to recruit patients. Interested patients receive an informational letter explaining the study aims and procedures. After 1 week, the patient is approached by telephone to provide further information, answer questions and check the (remaining) in- and exclusion criteria. Eligible patients who are willing to participate are invited to the study center to sign written informed consent and to undergo baseline measurements. Patients who choose not to participate in the EFFECT study are asked, but not required, to provide a reason for non-participation.

After baseline assessments, participants are randomly allocated (1:1) to either the exercise intervention or control group by central computerized randomization using a blocked computer-generated sequence, effectively blinding randomization. Randomization is stratified by study center and therapy line (maximum of two treatments received versus more). Due to the nature of the intervention, blinding of participants, local study nurses, or investigators to intervention assignment is not possible.

### Exercise intervention

The 9-month exercise program starts with a 6-month period where patients participate in a supervised, multimodal exercise program of 1 h twice a week. Thereafter, one supervised session is replaced by one unsupervised session for 3 months. Supervision is performed by an exercise professional (trainer). This can be an exercise physiologist or (oncology) physiotherapist who is trained by the study team to ensure a safe and standardized execution of the exercise program. Exercise sessions are offered at community or hospital-based fitness centers, physical therapy practices, gyms or personal training facilities throughout the catchment areas of the recruiting sites and close to the patients’ home address.

Each participant has an intake session in order to individualize the standard exercise prescription to their specific needs. The multimodal exercise program comprises aerobic-, resistance- and balance components. In case of physical limitations, an adapted exercise program is provided. Questions of local trainers can be presented to the EFFECT exercise expert board (including oncologists and exercise professionals from all study centers).

If training facilities are closed due to local COVID-19 regulations or if participants do not feel safe to visit them, an alternative live-online training program is provided by the trainer that includes the same components as the face-to-face exercise program. If needed, the study team provides participants with the required training equipment (e.g., resistance bands and free weights for resistance training; a cycle ergometer or an aerobic step for aerobic training). Prior to the start of the live-online program, the trainer conducts one individual face-to-face exercise session in which the exercises, training equipment, and training documentation are explained. During each session, the trainer closely monitors each participant to ensure that all exercises are performed correctly and safely.

#### Balance training

Each exercise session starts with 5 min of balance training. The balance exercise component includes exercises involving a variety of different stances (e.g., tandem stand) and can be progressed by including more challenging tasks. Progression depends on the subjective evaluation of both the trainer and participant.

#### Aerobic training

The aerobic training consists of a moderate-intensity continuous training (MICT) and progresses to high-intensity interval training (HIIT). The aerobic training protocol is individualized based on the patient’s fitness level using the Maximal Short Exercise Capacity (MSEC) and estimated W_peak_ as determined at baseline, 3 and 6 months post-baseline with the Steep Ramp Test (SRT; *see Study Outcomes*). Since the prescribed training load applies to the cycle ergometer, at least one training session per week should be performed on a cycle ergometer. The other training session can be performed on the rowing machine, treadmill, or elliptical/cross trainer, using the Borg scale to monitor intensity. For participants with bone metastases, aerobic training is preferably performed on the cycle ergometer or treadmill.

The exercise intensity is gradually increased during the exercise program (Table 1). The perceived intensity of the aerobic training is assessed using the Borg scale [13]. Since this is a vulnerable population and health status can deteriorate, the load should be decreased by 10% if the Rate of Perceived Exertion (RPE; 6-20) is higher than 15, whereas the load can be increased by 10% if the RPE is lower than 13.

**Table 1:** EFFECT exercise protocol

Week	Aerobic training (15 min)	Resistance training (35 min)
1–3	15 min MICT at 50–60% of W_peak_	Six exercises. Three sets per exercise^b^. The intensity is periodized, alternating between 10 and 12 reps with 70–75% of h1RM and 6 and 8 reps with 80–85% of h1RM every month.
4–14	Interval training^a^: 8 × 1 min at W_peak_, alternated with 1 min active rest at 30 Watts	
15–25	Interval training^a^: 3 × 3 min at 70% of W_peak_, alternated with 2 min active rest at 30 Watts	
26–36	Interval training^a^: 8 × 30 s at 65% of MSEC, alternated with 1 min active rest at 30% of MSEC	

*MICT* Moderate-Intensity Continuous Training, *MSEC* Maximal Short Exercise Capacity, *h1RM* hypothetical 1- Repetition Maximum

^a^All interval sessions start with a 3-minute warm-up at 30 Watts and conclude with a 3-minute cool-down

^b^Main exercises include the leg press, leg curl, leg extension, chest press, seated row, lat pulldown. Variations of these exercises are allowed and depend on the exercise modality

#### Resistance training

Resistance training consists of six exercises targeting the major lower and upper body muscle groups. The exercises can be delivered using the following modalities: machine-based, free-weights, or body-weight. All resistance exercises are individualized based on the patient’s fitness level, which is assessed by the trainer during the intake session using 12-repetition maximum (12-RM) muscle strength tests. Three sets per exercise are performed and the intensity is periodized (Table 1). To ensure a sufficient training load throughout the 9-month intervention period, the weights are continuously adjusted according to a progressive training protocol and in line with the periodization (if the participant’s health status permits) so that the predefined maximum number of repetitions is met as closely as possible. For participants with bone metastases, 12-RM testing is not performed for exercises that load regions with bone metastases (Table 2). Accordingly, adaptations to resistance exercises are shown in Table 2.

**Table 2:** Adaptations to the prescribed exercise program based on location of bone metastases

Metastases site	Resistance exercise^**a**^			Aerobic exercise		Flexibility
	Upper	Trunk	Lower	WB	NWB	Static
**Pelvis**	**√**	**√**	**√**^c^		**√**	**√**
**Axial skeleton (lumbar)**	**√**		**√**		**√**	**√**^d^
**Axial Skeleton (thoracic/ribs)**	**√**^b^		**√**	**√**	**√**	**√**^d^
**Proximal humerus**		**√**^b^	**√**	**√**	**√**	**√**^b^
**Proximal femur**	**√**	**√**	**√**^c^		**√**	**√**
**All regions**	**√**^b^		**√**^c^		**√**	**√**^d^

This table is adapted from Galvão et al. (2011) [14]

^a^Resistance exercises that load the affected region can be either omitted according this table or can be performed using a “start low, go slow” approach, depending on patient characteristics and the experience of the involved trainer. According to this approach, participants with bone metastases should start with low weights and more repetitions and increase weights gradually over time up to 10-12 repetitions if possible. Higher intensities (i.e., 6–8 repetitions with 80–85% of h1RM) should be avoided. Weights will be reduced if participants report pain during a resistance exercise or experience an increase in pain or pain medication since the last exercise session

√ = Target exercise region

^b^exclusion of shoulder flexion/extension/abduction/adduction and inclusion of elbow flexion/extension

^c^exclusion of hip extension/flexion and inclusion of knee extension/flexion

^d^exclusion of spine/flexion/extension/rotation

*WB* weight bearing (e.g., walking), *NWB* non-weight bearing (e.g., cycling)

#### Unsupervised exercise program

In addition to the supervised exercise program, participants are encouraged to be physically active for at least 30 min per day on all remaining days of the week. The participants are provided with an activity tracker (i.e., Fitbit Inspire HR) and an exercise app specifically designed for the EFFECT study.

As indicated above, during the last 3 months of the program, one supervised session is replaced by one unsupervised session of 60 min, supported by the exercise app. The app includes exercises that participants have learned during the supervised exercise program and can be performed at home using body weight, free weights, or resistance bands. All exercises are illustrated with simple animations and contain clear instructions. Participants receive instructions on the use of the exercise app from the study team. Participants are supported by the trainer to effectively use the exercise app and to successfully make the transition to unsupervised exercise.

#### Control group

Patients randomized to the control group receive standard medical care. They do not receive a structured exercise intervention as this is not yet part of routine care. In line with current physical activity guidelines, the control group participants are advised to avoid inactivity and to be as physically active as their health status allows [9]. They also receive an activity tracker.

### Study outcomes

Patients visit the clinical center for measurements at baseline and 3 and 6 months post-baseline. This includes assessments of physical fitness, body composition, and blood markers (Table 3). For patients undergoing chemotherapy, these assessments take place at least three days after chemotherapy infusion. The primary outcome assessment is at 6 months. All questionnaires are completed at baseline, 3, 6, and 9 months post-baseline (Fig. 1). Socio-demographic data are assessed at baseline with a study-specific questionnaire. Medical data are retrieved from the medical records. Personal data are coded and all data are handled in compliance with the General Data Protection Regulation (GDPR) (EU) 2016/679.

**Table 3:** Overview of all measurements in the EFFECT trial

		T0	T1	T2	T3
Outcomes	Instrument	Baseline	Month 3 ***± 14 days***	Month 6 ***± 14 days***	Month 9 ***± 21 days***
**Primary outcomes**					
**Cancer-related fatigue**	EORTC QLQ-FA12	X	X	X	X
**Health-related quality of life**	EORTC QLQ-C30	X	X	X	X
**Secondary outcomes**					
**Patient-reported outcomes**					
**Breast cancer-specific symptoms**	EORTC QLQ-BR45	X	X	X	X
**Depression and anxiety**	Patient Health Questionnaire (PHQ-4)	X	X	X	X
**Sleep problems**	Pittsburgh Sleep Quality Index (PSQI)	X	X	X	X
**Pain**	Brief Pain Inventory Short Form (BPI-SF), PainDETECT, Pain Catastrophizing Scale (PCS)	X	X	X	X
**Quality of working life**^**a**^	Quality of Working Life Questionnaire for Cancer Survivors (QWLQ-CS)	X	X	X	X
**Subjectively measured physical activity**	Modified version of the GODIN questionnaire	X	X	X	X
**Productivity loss**	Productivity Cost Questionnaire (iPCQ)		X	X	X
**Healthcare resources consumption**	Medical Consumption Questionnaire (iMCQ)		X	X	X
**Health-related quality of life**	EQ-5D-5L	X	X	X	X
**Urinary incontinence**	International Consultation on Incontinence Questionnaire -Urinary Incontinence Short Form (ICIQ-UI SF)^b^	X	X	X	X
**Satisfaction with exercise intervention**^**g**^	Self-developed questionnaire		X	X	X
**Physical measurements**					
**Physical performance**	5-times sit-to-stand, short Fullerton Advanced Balance (S-FAB) scale	X	X	X	
**Physical fitness**	Steep Ramp Test (SRT), endurance cycle test, handgrip- and leg strength test, Cardiopulmonary Exercise Testing (CPET)^c^, Isokinetic and isometric peak torque^d^	X	X	X	
**Objectively measured physical activity**	Physical activity tracker (Fitbit Inspire HR)	X	X	X	X
**Muscle thickness**^**e**^	Ultrasonography	X	X	X	
**Body composition**	Bio-impedance, DEXA^f^	X	X	X	
**Anthropometry**	• Body weight	X	X	X	
	• Waist and hip circumference	X	X	X	
	• Height	X			
**Resting heart rate and blood pressure**	-	X	X	X	
**Blood markers**	Plasma, serum, buffy coat and peripheral blood mononuclear cells	X	X	X	
**Socio-demographic and medical data**					
**Socio-demographic data**	Self-developed questionnaire	X			
**Medical history and concomitant diseases**	Medical records	X	X	X	X
**Cancer progress and treatment over the course of the study**	Medical records	X	X	X	X
**Cancer characteristics and treatment history**	Medical records	X	X	X	X
**Concomitant medication**	Medical records	X	X	X	X
**Adverse events**	Reports of patients, trainers, oncology nurses, physicians or medical records	X	X	X	X
**Overall and breast-cancer-specific survival and progression-free survival**	Medical records and/or cancer registry	Up to 5 years after the 9-month intervention period			

^a^Add-on measurement in the following clinical centers: UMCU, NKI, DKFZ, and ACU

^b^Add-on measurement in the following clinical center: ONK, UMCU, and NKI

^c^Add-on measurement in the following clinical center: KI and DSHS

^d,e^Add-on measurement in the following clinical center: KI

^f^Add-on measurement in the following clinical center: KI and ACU

^g^Only for the exercise group

### Primary outcomes

The primary outcomes of the EFFECT study are cancer-related physical fatigue and health-related QoL (HRQoL). Cancer-related physical fatigue is measured using the questionnaire of the European Organization for Research and Treatment of Cancer (EORTC) that is specifically developed and validated for assessing cancer-related fatigue (EORTC QLQ-FA12) [15]. The EORTC QLQ-FA12 is a 12-item questionnaire and assesses physical, cognitive, and emotional dimensions of cancer-related fatigue. Scores range from 0 to 100 with a higher score indicating higher levels of fatigue. HRQoL is measured using the EORTC QLQ-C30 summary score, which includes all original QLQ-C30 subscales excluding the global QoL score and financial difficulties score [16, 17]. Scores range from 0 to 100 with a higher score indicating a better HRQoL.

### Secondary outcomes

#### HRQoL, fatigue, anxiety, depression, sleep problems, and pain

Secondary outcome measures comprise the EORTC QLQ-C30/-BR45 functional and symptom scales and the other fatigue dimensions of the EORTC QLQ-FA12 (emotional, cognitive, and total fatigue scores). Anxiety and depression are assessed using the Patient Health Questionnaire for depression and anxiety (PHQ-4), which consists of two core anxiety items and two core depression items [18]. Sleep problems are assessed using the Pittsburgh Sleep Quality Index (PSQI), which contains 19 self-reported items assessing subjective sleep quality, sleep latency, sleep duration, habitual sleep efficiency, sleep disturbances, use of sleeping medication, and daytime dysfunction over the past month [19]. Pain is assessed using the Brief Pain Inventory Short Form (BPI-SF) and the Pain Catastrophizing Scale (PCS). The BPI-SF is a validated 11-item questionnaire and measures the severity of pain and its impact on daily functioning on a 0–10 numeric scale with the highest scores indicating worst pain and complete interference, respectively [20]. The PCS is a validated 13-item questionnaire designed to measure catastrophic thinking related to pain on a 0–4 scale with higher scores indicating a higher level of catastrophizing [21]. Neuropathic pain is assessed using the 13-item painDETECT screening questionnaire [22].

#### Resting heart rate and blood pressure

Resting heart rate and blood pressure are measured prior to the physical fitness and performance measurements (see Appendix II for details).

#### Physical fitness and performance

The order of the physical fitness and performance measurements is standardized. First, we assess functional performance and subsequently, MSEC, muscle strength, and aerobic capacity.

#### Functional performance

Originally, we planned to assess physical performance using the Short Physical Performance Battery [23]. However, due to the observation of ceiling effects in our first participants, we decided to replace this testing battery with the Short Fullerton Advanced Balance (S-FAB) scale [24] and the 5 times sit-to-stand test [23, 25]. The S-FAB scale measures static and dynamic balance during four tasks, including the tandem walk, standing on one leg, standing on foam with eyes closed, and stepping up onto and over a 6-inch bench. S-FAB tasks are rated on 0- (unable to complete task) to 4-point ordinal scale (independent task completion) with higher scores indicating better balance. The 5 times sit-to-stand test assesses functional strength of the lower limbs. The time (in seconds) required to perform 5 rises from the chair to an upright position as fast as possible is measured. This test is contraindicated for patients with spinal or pelvis metastases, who are unable to perform the test without impact on the spine/pelvis.

#### Maximal Short Exercise Capacity

MSEC is measured with the SRT using a cycle ergometer [26]. After 3 min of unloaded cycling, the test starts at 25 Watts and is increased by 2.5 Watts per second or 25 Watts per 10 s until exhaustion. Participants are instructed to cycle between 70 and 80 revolutions per minute (RPM). The test ends when cycling cadence drops below 60 RPM or when the participant experiences any pain or anxiety. After termination, the participant is asked to continue cycling at an easy cadence and with minimal load to recover. The outcome is registered as the highest achieved output in Watts and is referred to as MSEC. From the MSEC, peak Wattage (W_peak_) can be estimated using a regression equation [27]. Additionally, we record the RPE, time cycled, and heart rate at the end of the test as well as 1 and 2 min after termination.

#### Muscle strength

Upper body muscle strength is measured using a handgrip dynamometer (hydraulic Jamar®) with participants seated, their elbow by their side and flexed to the right angle (70°), and a neutral wrist position. Participants are asked to squeeze the dynamometer as hard as possible. Three measurements are performed on each hand and the best attempt of each hand is recorded. Lower body muscle strength is assessed using a leg-press hypothetical 1-repetition maximum (h1-RM) test according to a standardized protocol or an isokinetic dynamometer (IsoMed 2000®), unless the presence of bone metastases in the lower body prohibits safe testing. Measures of lower body muscle strength may differ between centers, but not within centers. For the h1-RM test, we record the highest weight that was successfully lifted for 12 repetitions and the corresponding h1-RM [28]. Maximal isokinetic peak torque (MIPT) is tested and recorded for each leg at 60°/s with the isokinetic dynamometer.

#### Aerobic capacity

Aerobic capacity is assessed using a constant-load exercise test to exhaustion on a cycle ergometer. The load is determined as 70% of the estimated W_peak_ derived from the SRT at baseline. If the SRT is terminated early and there are objective reasons to believe that a maximal MSEC was not achieved, 80% of the estimated W_peak_ is used as the load to avoid a ceiling effect of the test. Participants are instructed to maintain a speed of ~70 RPM. The test ends when cycling cadence drops below 60 RPM or when the participant experiences any pain or anxiety. After termination, the participant is asked to continue cycling at an easy cadence and minimal load to recover. The following parameters are recorded: time cycled in minutes, RPE, and heart rate at the end of the test as well as 1 and 2 min after termination.

#### Anthropometry

Anthropometric data (i.e., body weight, height, waist and hip circumference) are measured in light clothing without shoes (see Appendix II for details).

#### Body composition

Body composition (i.e., fat mass and fat-free mass) is measured prior to physical fitness testing by whole-body single frequency (50 kHz) bio-electrical impedance analysis (BIA). Patients are measured in a fasted state (no enteral intake for a minimum of 2 h) in a standing or lying position. Raw BIA data (i.e., reactance, resistance, and phase angle) are registered and estimates of fat mass and fat-free mass are obtained using the Kyle equation [29]. BIA devices may differ between centers, but patients within each center are measured consistently on the same device.

#### Blood markers

Plasma, serum, buffy coat and peripheral blood mononuclear cells (PBMC) are derived from whole blood samples (30 mL). In the 24 h prior to blood sampling, participants are instructed not to exercise vigorously or drink alcohol, and in the 2 h prior to blood sampling, they are asked to abstain from cigarettes, food, and drinks. Immediately after collection, blood samples are centrifuged and stored at -80°C at the local laboratory according to standardized procedures. Blood samples are transferred to the central biobank at the Karolinska Institutet for analysis after the last sample has been collected locally.

#### Physical activity

The Modified Version of the Godin-Shephard Leisure-Time Exercise Questionnaire and complementary questions on types and settings of exercise are used to measure self-reported physical activity levels [30, 31]. The Godin questionnaire is a 4-item questionnaire, including questions on the average frequency and duration one engages in mild, moderate, and vigorous aerobic activities and moderate-to-vigorous resistance exercises in bouts of at least 10 min during leisure time in a typical week. In addition, the Fitbit Inspire HR is used to objectively measure step count, heart rate, and physical activity minutes. Both intervention group and control group participants are asked to wear the activity tracker throughout the whole study period, but in any case during seven days after randomization and seven days before T1 (3 months), T2 (6 months), and T3 (9 months).

#### Cost-effectiveness

The EQ-5D-5L is used to measure health in five dimensions, including mobility, self-care, usual activities, pain/discomfort, and anxiety/depression, using 5 levels of severity [32]. This questionnaire is used to calculate quality adjusted life years (QALYs) during follow-up.

The actual costs incurred within both study arms will be compared until nine months after randomization. The cost-effectiveness analysis (CEA) will be done from a societal perspective, including healthcare costs, patient and family costs, and productivity costs. Participants are asked to complete questionnaires on these different types of costs. A health care use questionnaire was developed before the start of the study, based on the iMTA Medical Cost Questionnaire (iMCQ) [33], including cost categories that are deemed relevant for patients with mBC. Productivity losses are measured using the Productivity Cost Questionnaire (iPCQ) [34]. These questionnaires are completed 3, 6, and 9 months post-baseline.

#### Adherence

Adherence incorporates both attendance at the supervised exercise sessions and compliance with the prescribed exercises according to protocol. For each scheduled session, the trainer documents attendance and compliance in a case report form (see Appendix II for details).

#### Satisfaction

At 3, 6, and 9 months, we assess satisfaction with the supervised exercise program, the exercise trainer, the activity tracker, and supporting exercise app by means of a study-specific questionnaire.

#### Disease progression and survival

Participants will be followed for disease progression and survival for 5 years beyond the 9-month study period. Information on disease progression and (all-cause and breast cancer specific) death is retrieved from medical records and/or the cancer registry.

#### Add-on measurements

The following measurements are completed at some clinical centers (Table 3): quality of working life, urinary incontinence, cardiopulmonary exercise testing, whole body dual-energy X-ray absorptiometry, isokinetic and isometric peak torque, and muscle thickness. See Appendix II for details.

#### Safety

All (serious) adverse events ((S)AE) related to exercise or study measurements are recorded. Participants in both groups are asked by the study personnel about exercise- and study measurement-related (S) AEs in a standardized manner during all follow-up visits. In addition, participants allocated to the exercise group are asked by their trainer, before and after each supervised session, whether any potentially exercise-related (S) AEs occurred during or since the last exercise session. The trainers are instructed to contact the study team if any (S) AE occurred. All (S) AEs are recorded by the study team and SAEs are reported to the accredited ethical committee that approved the protocol, according to the requirements of that ethical committee.

### Sample size

An improvement of either or both of the primary outcomes, i.e. cancer-related physical fatigue or HRQoL, from baseline to 6 months post-baseline relative to control is of relevance. To adjust for multiple testing, the Bonferroni-Holm method will be used. Based on results from 6 randomized exercise trials in patients with breast cancer receiving adjuvant treatment, we anticipate an effect size of 0.35, which has been found in a pooled analysis [35]. With *n*=139 patients per group (*n*=278 in total), for each endpoint separately a mean standardized effect size of at least 0.35 can be detected with an analysis of covariance (ANCOVA) adjusted for baseline values of the outcome with a power of at least 78% or 82% at a (nominal) two-sided significance level of 2.5%, assuming a correlation between pre- and post-intervention levels of Rho=0.3 or Rho=0.4, respectively [36]. However, the probability of at least one of the two tests corresponding to the two primary outcomes to yield a significant result, if both alternative hypotheses are true, is higher. In addition, taking repeated measures into account using mixed models might further increase the study power. To account for a potential drop-out rate of approximately 20%, a total number of *n*=350 patients will be enrolled into the study (*n* = 175 per study arm). This sample size will also facilitate exploratory moderator and subgroup analyses to better understand which patients benefit most from the exercise program.

### Statistical analysis

Descriptive statistics will be used to characterize the study population at baseline. Questionnaire scores will be calculated according to published scoring manuals. Analyses will be performed according to the intention-to-treat principle. For the primary outcomes, mixed linear regression models will be used to assess exercise effects on physical fatigue and HRQoL separately, while taking the hierarchical structure of the data into account. Models will be adjusted for the baseline value of the outcome and stratification factors (i.e., center and therapy line). To adjust for multiple testing, the Bonferroni-Holm method will be used to maintain an overall alpha level of 5%. The same analysis will be performed for secondary outcomes. The detailed statistical analysis plan is included in the IRB study protocol.

In the economic evaluation, the balance between costs and effects of both study arms will be assessed and compared up until nine months after randomization. Results of both cost and effect measurement will be integrated using cost-effectiveness and cost-utility analyses. In the cost-utility analysis, efficiency is expressed in terms of costs per QALY. In the cost-effectiveness analysis, costs per unit of change in the two primary outcome measures will be estimated. Finally, incremental costs and incremental effects, expressed in a ratio (ICER) will be estimated. A probabilistic uncertainty analysis using bootstrapping will be performed.

To assess the potential effect of exercise on progression-free, overall, and breast cancer-specific survival, we will use Cox proportional hazard regression models stratified by center and adjusted for pre-specified prognostic factors, including therapy line, type of baseline metastases, age, and time since diagnosis of first metastases.

Potential moderators of the exercise effect will be explored (e.g., age, baseline fitness level, type of therapy, and location of metastases). In addition, mediation analyses will be performed to explore potential underlying mechanisms of exercise effects (e.g., blood markers).

Missing data due to disease progression or mortality will certainly occur, which could be Missing Not At Random (MNAR) if the exercise intervention also has an effect on progression and survival. To explore potential bias, sensitivity analyses according to EMA recommendations will be conducted using approaches that investigate different MNAR scenarios such as a pattern mixture model.

### Data capturing and monitoring

Castor®, a cloud-based clinical data management platform, is used for randomization and data capture. Castor is also used to send out questionnaires to all participants. Validity of the data is checked by an independent monitor.

## Discussion

During palliative cancer treatment, many patients experience cancer- and treatment-related side effects that impair daily life activities and negatively affect QoL. Exercise during and after curative cancer treatment is a proven strategy to minimize these side effects. Since the majority of the available literature is in early stage cancers [9], results may not be directly generalizable to patients with advanced cancers due to the nature of the disease, differences in treatments, and the higher risk of disease progression. In the current trial, we are investigating the (cost-)effectiveness of a 9-month exercise program in mitigating fatigue and maintaining or enhancing QoL in patients with mBC.

Due to the COVID-19 pandemic, we were forced to make some (temporary) changes to the original study protocol and offer live-remote training. These changes were approved by the Medical Ethics Committee of the University Medical Center Utrecht and local ethical committees and are being reported according to the CONSERVE-SPIRIT guidelines [37]. Furthermore, the local COVID-19 regulations might result in a delay in recruitment or a cancelation of follow-up visits, since patients are not allowed or willing to visit the study center for study-related activities. All COVID-19-related changes will be reported alongside the primary results of our study.

In this study, we decided to include any patient who is currently being treated for mBC with at least a 6-month life expectancy, independent of the number of treatments received. We are aware that this approach will result in a heterogeneous, but representative, group of patients. Since the line of treatment might affect our outcomes, we stratify our randomization based on the therapy line (maximum of two treatments received versus more). Note, we decided to not include patients with oligometastatic breast cancer who are treated with curative intent

Meta-analyses of exercise-oncology trials have suggested that supervised exercise programs and programs that involve both aerobic and resistance exercises are most effective [38, 39]. In general, compliance with this exercise prescription is reported to be above 80% [40]. For patients with mBC, we anticipate that adaptations will be required, since we expect patients to present with a high disease burden and fluctuating health and performance status. This involves regular monitoring of pain and fatigue and will result in personalized adaptation of the exercise prescription. In this way, exercise intensities can be increased or decreased based on the self-reported severity of exertion. We think of such ad-hoc adaptations to the pre-planned training schedule as appropriate exercise prescription for this specific population, and not as evidence of lack of feasibility or poor intervention fidelity. A guide for selecting the appropriate exercise intensity and volumes is provided to all trainers. To improve gait stability and hence activities of daily living, balance exercises are included in the program. Balance exercises have also been suggested to improve limitations due to chemotherapy-induced peripheral neuropathies [41–44].

The skeleton is the most common site for distant metastases in breast cancer. In addition, patients can suffer from osteoporosis due to treatment with aromatase inhibitors [45]. Given the expected increased risk of skeletal-related events, including pathological fracture and spinal cord compression, exercise is often underutilized by trainers [46–48]. A recent systematic review showed that exercise is safe (no exercise-related SAEs were reported) and feasible for patients with bone metastases if it includes a supervised component [11]. In the current study, all exercise sessions are supervised by a trainer specially trained by the study team to ensure safe execution of the exercise program. Based on findings of the aforementioned review [11] and previous research in patients with advanced cancer [49], we provide all trainers with specific instructions on adaptations that need to be made to the prescribed exercise protocol in case of bone metastases. Nevertheless, exercise in patients with mBC remains challenging and additional adaptations to the exercise program may be required. Therefore, we have created an EFFECT exercise expert board that will respond to questions and request for additional information from the trainers. Additionally, members from all recruiting sites meet twice weekly to discuss exercise-related questions

In the curative setting, exercise programs from 12 weeks or longer are reported to be effective [9]. In the metastatic setting, patients generally receive continuous treatment. Therefore, we offer a longer intervention, i.e., a 6-month exercise program with 2 supervised session per week, followed by 3 months with one supervised exercise session replaced by one unsupervised session, which will be supported by an activity tracker and exercise app. Additionally, the trainer encourages the participant to be active in daily life. We hypothesize that the transition from supervised to unsupervised exercise sessions will help participants to maintain a physically active lifestyle beyond the period of study participation.

In exercise intervention studies, results might be affected by low compliance to the intervention and contamination of the control group. In order to increase attendance and compliance, our exercise sessions are individualized and supervised by specially trained and experienced trainers, and are offered close to the patients’ home. In addition, we closely monitor adherence and compliance in the study. Contamination (i.e., adoption of something similar to the intervention by the controls) is reported in 37% of all exercise-oncology trials. We took recommended measures to decrease the risk of contamination, i.e., we include relatively inactive patients, clearly explain the randomization procedure to avoid disappointment when being randomized to the control condition, and provide general exercise advice and an activity tracker to the control group [50]. While controls' physical activity level might be increased by providing the activity tracker, it has been shown that providing something to the control group decreases the risk of drop-out and of contamination [50]. Patients who consent to participate in an exercise intervention trial are generally willing to exercise, and activity trackers have been observed to provide a low-level stimulus to engage in physical activity. Thus, the EFFECT trial will assess whether the exercise intervention is significantly better than a simple low-level physical activity stimulus.

In conclusion, in the EFFECT study, we are investigating the effects of a supervised exercise program in patients with mBC on physical fatigue and QoL, as well as a range of other patient-reported, biomedical and objective health outcomes. If exercise during palliative treatment of patients with mBC is proven to be (cost-)effective, implementing exercise as an integral and standardized component of palliative care would be a logical next step. The results of our study can also inform international guidelines with respect to the role of exercise in improving the QoL of patients with advanced stages of disease and reducing cancer- and treatment-related side effects.

### Trial status

Protocol version number and date: version 4.0 dd 25-05-21

Date start recruitment: 08-01-2020

Approximate date end of recruitment: 01-09-2022

## Supplementary Information

**Additional file 1: Appendix I.** Organizational aspects of the trial.

**Additional file 2: Appendix II.** Add-on measurements.

## Data Availability

The consent materials are available from the corresponding author on request. The datasets used and/or analyzed during the current study are available from the corresponding author on reasonable request.

## Abbreviations

ANCOVA: Analysis of covariance
BIA: Bio-electrical impedance analysis
BMI: Body mass index
BPI-SF: Brief Pain Inventory Short Form
CEA: Cost-effectiveness analysis
CPET: Cardiopulmonary exercise testing
DEXA: Dual-energy X-ray absorptiometry
ECOG: Eastern Cooperative Oncology Group
HIIT: High-intensity interval training
ICIQ-UI SF: International Consultation on Incontinence Questionnaire - Urinary Incontinence Short Form
iMCQ: Medical Cost Questionnaire
iPCQ: Productivity Cost Questionnaire
HRQoL: Health-related quality of life
mBC: Metastatic breast cancer
MICT: Moderate-intensity continuous training
MNAR: Missing Not At Random
MSEC: Maximal Short Exercise Capacity
PBMC: Peripheral blood mononuclear cells
PCS: Pain Catastrophizing Scale
PHQ: Patient Health Questionnaire
PSQI: Pittsburgh Sleep Quality Index
QALY: Quality adjusted life year
QoL: Quality of life
RF: Rectus femoris
RPE: Rate of perceived exertion
RPM: Revolutions per minute
RM: Repetition maximum
(S)AE: (Serious) adverse event
S-FAB: Short Fullerton Advanced Balance scale
SRT: Steep Ramp Test
VL: Vastus lateralis
