# Supplementary material for: Design of a multinational randomized controlled trial to assess the effects of structured and individualized exercise in patients with metastatic breast cancer on fatigue and quality of life: the EFFECT study
Source: Trials. 2022 Jul 29;23:610. doi: 10.1186/s13063-022-06556-7 (PMC9335464; doi:10.1186/s13063-022-06556-7)
Supplement: Supplementary file 1 — Additional file 1: Appendix I. Organizational aspects of the trial. [file 13063_2022_6556_MOESM1_ESM.docx]

**Appendix I**

Here, we provide more details with respect to organization of the trial.

Sponsor of the EFFECT study

University Medical Center Utrecht

Julius Center for Health Sciences and Primary Care

Heidelberglaan 100, 3584 CX Utrecht, The Netherlands
PI Prof. Dr. Anne May, Professor of Clinical Epidemiology of Cancer Survivorship

Project management

The day-to-day operational project management, coordination and oversight of the EFFECT trial is the responsibility of Prof.Dr. May, supported by researchers of the UMC Utrecht.

Study management

A Clinical Research Organisation (Julius Clinical// https://www.juliusclinical.com/) is responsible for the study management of the EFFECT trial. This includes the following activities: obtaining regulatory and ethical approval, site contracting, training of monitors, data management, processing and reporting of SAE’s, and monitoring at sites.

Steering committee (consisting of all WP leaders)

Decisions which concern changes in strategy are made by the steering committee of EFFECT. The committee consists of the EFFECT WP leaders and/or at least one representative of the participating centers. The steering committee is chaired by Prof. Dr. May and established under mutual agreement with the entire consortium. Decisions of the committee are made first and fore most on consensus making.

Data-management

Study data will be captured using an eCRF (Castor®). Castor® is compliant with all relevant regulations, such as ICH E6 Good Clinical Practice and the General Data Protection Regulation (GDPR). Required data for this study are to be obtained from the participant’s medical records/source documents or by direct entry, where the information was first recorded and then entered into the eCRF. Data from the eCRF will be encoded and stored in a study database. Only authorised site staff will be allowed to enter data into the eCRF and make changes to eCRF data. Additionally, data will be obtained from online questionnaires filled in by the participants.

The eCRFs will be reviewed by a monitor from the CRO for completeness and accuracy as described in the Monitoring Plan of the study.

Data-management details are described in a data-management plan.

Data Safety Monitoring Board (DSMB) and interim analyses

Not applicable. We consider a DSMB not necessary since this is a low risk trial. The safety of physical exercise has been shown in previous studies in breast cancer survivors.

No interim analyses are planned.

Protocol amendments

Any modifications made to the protocol after receipt of the Regulatory Authorities / Ethics Committees approval will be re-submitted in accordance with local procedures and regulatory requirements. Modifications or new information that might change patients willingness to participate, will be communicated to the included patients.

Dissemination policy

The investigators will inform all interested participants about the main results of the study. The results of the study will be reported in peer reviewed international journals, national non-peer reviewed journals, e.g., from patient organisations and professional associations, and presented on (inter)national conferences. Also, policy regulators, healthcare providers and other important stakeholders will be informed on the outcomes of the EFFECT study .
