# Supplementary material for: Design of a multinational randomized controlled trial to assess the effects of structured and individualized exercise in patients with metastatic breast cancer on fatigue and quality of life: the EFFECT study
Source: Trials. 2022 Jul 29;23:610. doi: 10.1186/s13063-022-06556-7 (PMC9335464; doi:10.1186/s13063-022-06556-7)
Supplement: Supplementary file 2 — Additional file 2: Appendix II. Add-on measurements. [file 13063_2022_6556_MOESM2_ESM.docx]

**Appendix II**

Here, we provide more details with respect to the measurement of blood pressure, anthropometry and adherence.

*Blood pressure*
Participants’ resting heart rate is measured after a five minute rest in supine position. Blood pressure is measured on both arms with the higher pressure used as reference when inconsistencies between arms exist. Participants with severe hypertension at rest (systolic blood pressure >180 mmHg and/or diastolic blood pressure >110mmHg) require further medical evaluation prior to proceeding with physical fitness testing.

*Anthropometry*
To measure body weight, we use a calibrated analogue or digital weight scale, depending on study center. Height is measured using a wall mounted tape measure. The same weight scale and tape measure is used for all measurements of an individual participant. Body Mass Index (BMI) is calculated as weight in kilograms divided by height in meters squared (kg/m^2^).

Waist and hip circumference are measured halfway between the lower ribs and iliac crest using a non-elastic measuring tape, and at the largest circumference between waist and thigh, respectively. Measurements are taken in duplicate to the nearest 0.5 cm. If the difference between the two measurements is greater than 2 cm, a third measurement is taken and the mean of the two closest measurements is calculated. The waist-to-hip ratio is calculated by dividing the waist circumference by the hip circumference.

*Adherence*To monitor compliance, the trainer records peak and mean heart rate, load (in Watts) and duration of the aerobic training as well as weights and number of repetitions for the resistance training. Reasons for missed exercise sessions or non-compliance are documented. Every 2 weeks, this documentation will be sent to the researchers for monitoring. In addition to this documentation, physical activity data of the activity tracker and the exercise app provides insight into compliance to both the supervised and unsupervised exercise sessions. Moreover, during the intervention period each exercise expert is visited at least once by a researcher to ensure proper performance of the exercise protocol.

The following measurements are added to some clinical centers.

***Quality of working life and urinary incontinence***

The Quality of Working Life Questionnaire for Cancer Survivors (QWLQ-CS) is used to evaluate the quality of working life.^1^ The International Consultation on Incontinence Questionnaire Urinary Incontinence Short Form (ICIQ-UI SF) is used to assess the prevalence, frequency, perceived cause of urinary incontinence, and its impact on daily life.^2^

***DEXA***

A whole body dual-energy X-ray absorptiometry (DEXA) scan is used to determine regional and whole body lean and fat mass. Additionally, trunk adiposity, visceral fat and adipose indices are assessed. Scans are performed in a fasted state (no enteral intake for a minimum of 2 hours) and based on standard procedures.

***Isokinetic and isometric peak torque***

Maximal isokinetic and isometric strength of the legs is assessed using an isokinetic dynamometer at 60°/s and 180°/s. After a warm-up consisting of 10 knee extensions, participants perform two sets of two maximal efforts at each velocity with 30 seconds rest between sets of the same speed and 2 minutes rest between different speeds. The highest peak torque value is recorded. After 2 minutes of rest, maximal isometric torque is measured at a knee angle of 120°. Participants are instructed to push twice with maximal effort for approximately 5 seconds with 60 seconds of rest between the two attempts. The best score in a 1 second window defines peak isometric torque.

***Cardiopulmonary exercise testing* *(CPET)***

Cardiorespiratory fitness is determined by CPET on a bicycle ergometer with continuous breathing gas analysis. The CPET protocol starts at 20 Watts and gradually increases with 10 Watts/minute until exhaustion or symptom limitation. Participants are instructed to cycle at 60-80 RPM. The test is terminated when RPM drops below 60 and concludes with a 3-minute cool down at 20 Watt. During the test, information is provided through continuous 12-lead electrocardiography, heart rate, oxygen saturation and blood pressure monitoring as well as breath-by-breath analysis. Peak heart rate and peak oxygen consumption (VO_2peak_) are determined based on the highest 20 seconds average values during or immediately post-exercise. Maximal respiratory exchange ratio is determined based on the highest 20 seconds average values during exercise. In addition, peak power output is assessed and RPE is recorded every 2 minutes and at exercise termination. Ear lobe blood samples for the determination of peak blood lactate concentration are taken before the test, at termination and after 3 minutes of recovery.

***Muscle thickness***

Thickness of both m. rectus femoris (RF) and m. vastus lateralis (VL) are measured using ultrasonography while the participant is resting in supine position with the knees fully extended. The scanning site is located at 2/3 of the distance between the anterior superior iliac spine and the superior pole of the patella. Distance is measured in both legs and recorded. The transducer is placed transversally to the thigh with the ultrasound transducer oriented on a 90° angle to the muscle bundles. RF thickness is measured first, followed by VL, as the distance between superficial and deep aponeuroses. Two separate pictures are taken. If the values differ >3%, a third image is obtained. Pictures are considered valid when the normal curved shape on the superficial part of the muscle is clearly visible. Echogenicity (expressed as grayscale values; from 0 to ~255 in arbitrary units) of RF and VL is assessed in saved pictures.

**References**

1. de Jong M, Tamminga SJ, de Boer AGEM, Frings-Dresen MHW. Quality of working life of cancer survivors: development of a cancer-specific questionnaire. *J Cancer Surviv*. 2016;10(2):394-405. doi:10.1007/s11764-015-0485-4

2. Avery K, Donovan J, Peters TJ, Shaw C, Gotoh M, Abrams P. ICIQ: a brief and robust measure for evaluating the symptoms and impact of urinary incontinence. *Neurourol Urodyn*. 2004;23(4):322-330. doi:10.1002/nau.20041
